# Combination of serum RASSF1A methylation and AFP is a promising non-invasive biomarker for HCC patient with chronic HBV infection

**DOI:** 10.1186/s13000-015-0317-x

**Published:** 2015-08-04

**Authors:** Xueyan Dong, Hui He, Weiying Zhang, Daojun Yu, Xianjun Wang, Yueming Chen

**Affiliations:** Department of Laboratory Medicine, Hangzhou First People’s Hospital, 310006 Hangzhou, China; The Second Clinical Medical College, Zhejiang Chinese Medical University, 310053 Hangzhou, China

**Keywords:** Hepatocellular carcinoma, RAS association domain family 1A, Methylation

## Abstract

**Background:**

Hypermethylation of the promoter region of the RAS association domain family 1A gene (RASSF1A) occurs widely in hepatocellular carcinoma (HCC) tissues. While the diagnostic performance of the use of RASSF1A methylation as a serum or plasma marker in patients with HCC has varied largely in the literature,we confirmed the clinical application value of serum RASSF1A methylation for HBV related HCC in this study.

**Methods:**

A total of 584 participants were recruited into this study, including 190 patients with HCC, 114 patients with liver cirrhosis (LC), 120 patients with chronic hepatitis B (CHB) and 160 healthy individuals. Serum RASSF1A methylation was determined by the MethyLight method. In addition, we followed up 43 HCC patients who were unable to undergo surgery for 24 months.

**Results:**

Serum RASSF1A methylation occurred significantly more frequently in patients with HCC (122/190, 64.2 %) than in patients with LC (20/114, 17.5 %), patients with CHB (6/120, 5.0 %) and in healthy individuals (0/160, 0) (*P* < 0.001); moreover, it allowed for the discrimination of patients with HCC from those with CHB with an areas under the ROC curves (AUC) of 0.796 (64.2 % sensitivity and 89.8 % specificity). Furthermore, the AUC for the combination of serum RASSF1A methylation and AFP level (≥20 ng/L) was 0.876 (80.9 % sensitivity and 93.4 % specificity). Serum RASSF1A methylation positive in patients with HCC was associated with more malignant clinical characteristics and a worse overall survival (OS) (*P* < 0.05).

**Conclusion:**

Serum RASSF1A methylation demonstrated a satisfactory value for in the diagnosis of HBV related HCC, and could predict clinical progression and prognosis. In addition, our findings suggested that the combination of serum RASSF1A methylation and AFP level may be a promising non-invasive biomarker for the discrimination of patients with HCC from those with CHB.

**Virtual slides:**

The virtual slides for this article can be found here: http://www.diagnosticpathology.diagnomx.eu/vs/13000_DPAT-D-15-00090.1

## Background

HCC (Hepatocellular carcinoma, HCC) is one of the high incidence and mortality malignant tumors in the world. Chronic hepatitis B virus (HBV) infection is an important etiological factor of HCC in Southeast Asia. The estimated overall HBV carrier prevalence among Chinese aged 1 to 59 years was 9.75 %, and the prevalence in difference provinces in China ranged from 4.49 to 17.85 %, which has led to a large number of individuals who are at risk for HCC [1]. At present, a serum α-fetoprotein (AFP) assay is widely used, but it has a relatively low sensitivity and specificity, and thus its clinical value is limited. Depending on currently clinical diagnostic tools, the patients with HCC may not undergo the most effective treatment as most of them have already progressed to advanced stages by the time they are diagnosed. Therefore, there is an urgent need for the identification of effective diagnostic and prognostic biomarkers for HCC.

Aberrant DNA methylation is one of the most common characteristics of malignant cells. Some tumor suppressor genes and other pivotal genes that regulate cell signaling pathways are frequently silenced due to the promoter methylation in tumor tissues; these changes have been tested and shown to be sensitive and specific markers for HCC [2–4]. However, the use of tumor tissues to test for promoter methylation is invasive and thus impractical for HCC screening in high-risk populations. Several studies have shown that the detection of DNA promoter methylation in serum or plasma is highly consistent with its presence in tumor tissues [5], which suggests that the gene promoter methylation in the serum might be a promising non-invasive biomarker for tumor diagnosis.

The RAS association domain family 1A gene (RASSF1A) is a tumor suppressor gene that is located in the 3p21.3 region and is an important member of the RAS signaling pathway. It plays a critical role in apoptosis cell cycle, microtubule stability, cell adhesion and migration [6]. The RASSF1A gene has been concerned and studied intensively for its tumor suppression, and hypermethylation in the promoter region is suspected as the main mechanism of silencing that is observed widely in human malignancies, including HCC tissues [7, 8]. Although its diagnostic value in serum, plasma or the other body fluids of patients with HCC has been studied, large discrepancies exist in regards to its diagnostic performance, such as the sensitivity (0.27 to 0.94) and specificity (0.38 to 0.95) [9].

The aim of this study was to explore whether the promoter methylation status of RASSF1A as detected by MethyLight assay is altered in the serum of patients with HBV-related HCC compared with age- and gender-matched patients with chronic hepatitis B (CHB), liver cirrhosis (LC) and healthy individuals. In addition, we evaluated the potential relationship between serum RASSF1A methylation status and the clinicopathological features and overall survival (OS) of patients with HCC. Finally, we assessed whether serum RASSF1A methylation was a satisfactory biomarker for HCC patients with chronic HBV infection.

## Methods

### Subjects

A total of 584 participants who visited Hangzhou First People’s Hospital from June 2011 to June 2013 were enrolled in this study. They were divided into four age- and gender-matched groups (HCC, LC, CHB patients and healthy subjects). In all, 190 patients with HCC had been diagnosed by serum AFP level, liver ultrasound, computed tomography (CT), and magnetic resonance imaging (MRI). Those who met the diagnostic criteria for HCC, which was confirmed by histological examination were enrolled. These HCC patients had not received any preoperative radiotherapy or chemotherapy. 114patients with LC were diagnosed by liver ultrasound, CT, and MRI, and exhibited accompanying portal hypertension and hypersplenism. 120 patients with CHB met the diagnostic criteria based on the guidelines for the prevention and treatment of chronic hepatitis B (2010 version) by the Chinese Society of Hepatology and Chinese Society of Infectious Diseases of the Chinese Medical Association, and did not have LC and HCC as detected by liver ultrasound. 160 healthy individuals were obtainde from Physical Examination Center of Hangzhou First People’s Hospital.

In addition, the patients with HCC, LC and CHB were HBV surface antigen (HBsAg)-positive in the serum, and had not undergone anti-viral treatment. The participants who presented with other liver diseases, such as autoimmune hepatitis, alcoholic hepatitis and other types of hepatitis virus infections were excluded from this study. The study protocol was approved by the Ethics Committee of Hangzhou First People’s Hospital.

### Serum samples collection and DNA extraction

Two milliliters of serum was isolated after centrifugation at 3000 rpm for 10 min and was then centrifuged at 12,000 rpm for 5 min. One milliliter of serum was isolated carefully and stored at −80 °C until use. Serum DNA was extracted by a serum DNA extraction kit (GenMagBio Biotechnology Co., Ltd., Beijing, China) following the protocol recommended by the manufacturer.

### Sodium bisulfite modification

Serum DNA was modified by sodium bisulfite treatment and purified using the EpiTect Bisulfite Kit (Qiagen, Hilden, Germany) following the protocol recommended by the manufacturer.

### DNA methylation assay

The methylation status of RASSF1A was examined using methylation-specific quantitative PCR (MethyLight). The modified DNA samples were amplified with specific primers and Taqman probes. The sequences of the primers and probes for RASSF1A and β-actin (ACTB) were previously described [10, 11]. The PCR mixture contained 1 μl bisulfite-treated DNA, 0.15 μl of each primer (10 uM), 0.1 μl of each probe (10 uM), 9.6 μl nuclease-free water, and 10.0 μl 2 × PCR Buffer (Toyobo Co., Ltd, Japan), which consisted of Taq DNA polymerase, reaction buffer, and a deoxynucleotide triphosphate mixture, in a final volume of 20 μl. The PCR was performed for RASSF1A and ACTB in parallel in an ABI 7500 Sequence Detection System (Life Technologies, USA). The PCR program included an initial denaturation step at 95 °C for 3 min, followed by 45 cycles of denaturation at 95 °C for 10 s, and annealing at 60 °C for 1 min. Genomic DNA from healthy individuals, which was treated with SssI methylase and sodium bisulfite *in vitro*, was used as a positive control. DNA from healthy human blood was used as a negative control. Water without DNA served as a control for contamination was included in each assay. Serum RASSF1A methylation is presented as the Percentage Methylated Reference (PMR) [12]. A PMR ≥ 4 % would be classified as positive, while a PMR < 4 % would be classified as negative; these values have been validated in the literature as the standard cut-off values [13–16].

### Statistical analysis

The Mann–Whitney test was used to study the differences in the non-parameter variables. The Correlation between serum RASSF1A methylation and clinicopathologic parameters was determined using the *χ*2 test. Overall survival analysis was performed with the Kaplan-Meier analysis and log-rank test Receiver-operating-characteristic (ROC) curves were constructed and the areas under the ROC curves (AUCs) were calculated to determine the feasibility of using serum RASSF1A methylation and/or AFP level as a biomarker for HCC. A value of *P* < 0.05 was considered statistically significant. Data analyses were performed with SPSS software (version 15; SPSS Inc., Chicago, USA).

## Results

### Demographics

The baseline characteristics of the enrolled participants are shown in Table 1. Of those, the serum levels of alanine aminotransferase (ALT) and total bilirubin (TBIL) were obviously higher in cases of HCC, LC and CHB compared with the healthy controls (*P* < 0.05), whereas the albumin (ALB) and platelet (PLT) levels were higher in the healthy controls (*P* < 0.05). The frequency of a serum AFP level ≥20 ng/L was highest in patients with HCC followed by patients with CHB and patients with LC (*P* <0.05).

**Table 1:** The baseline clinical data of 584 subjects

Characteristics	HCC (*n* = 190)	LC (*n* = 114)	CHB (*n* = 120)	Health (*n* = 160)
Age (y)	52.32 ± 18.63	54.65 ± 15.39	54.65 ± 15.33	52.12 ± 16.76
Male	136 (71.58)	76 (66.67)	84 (70.00)	110 (68.75)
ALT (U/L)	62.82 ± 46.27	71.19 ± 32.40	90.81 ± 60.23	24.62 ± 16.28
ALB (g/L)	33.39 ± 14.57	30.18 ± 11.95	38.13 ± 8.26	40.13 ± 4.67
TBIL (μmol/L)	26.54 ± 19.83	76.49 ± 42.35	50.28 ± 46.03	9.81 ± 6.73
PLT (×10^9^/μL)	15.65 ± 13.07	11.36 ± 9.81	16.85 ± 13.49	19.04 ± 10.27
HBeAg (+)	96 (50.52)	60 (52.63)	68 (56.67)	0 (0)
HBV DNA (log_10_ copies/mL)	5.93 ± 1.86	5.78 ± 1.29	5.87 ± 1.13	0
AFP (≥20 ng/L)	118 (62.11)	62 (54.39)	32 (26.67)	0 (0)

There were significant differences in the levels of ALT, ALB, TBIL, PLT and AFP in the HCC, LC and CHB groups (*P* < 0.05). All data are presented as mean ± standard deviation or n (%)

*HCC* hepatocellular carcinoma, *LC* liver cirrhosis, *CHB* chronic hepatitis B; *ALT* alanine aminotransferase, *ALB* albumin, *TBIL* total bilirubin, *PLT* blood platelet, *HBeAg* HBV e-antigen, *AFP* α-fetoprotein

### Methylation status of RASSF1A in the serum

**Fig. 1:**
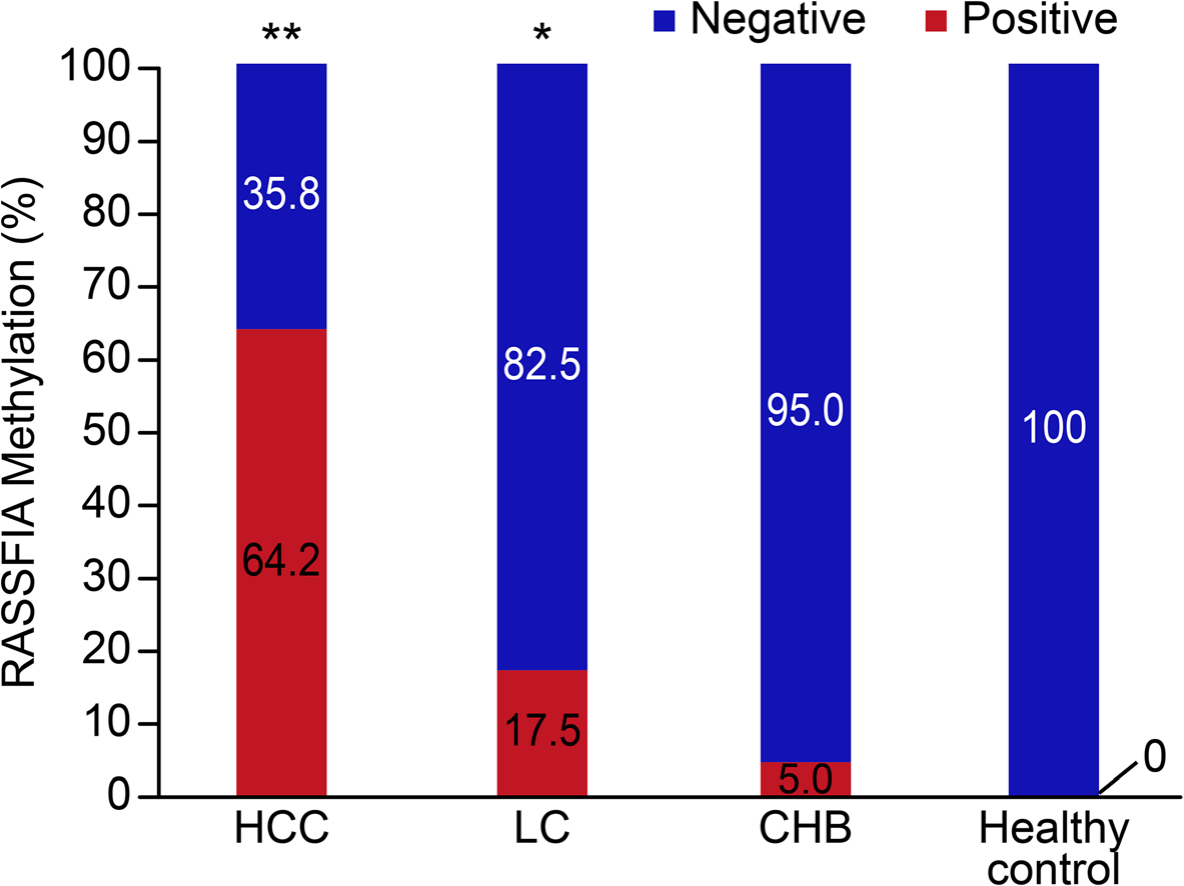
The rate of positive serum RASSF1A methylation in the HCC, LC, CHB and healthy control groups. The positive rate was 64.2 % (122/190), 17.5 % (20/114), 5.0 % (6/120) and 0 (0/160), respectively; *P* * < 0.01 versus CHB; *P* ** < 0.001 versus LC, CHB

Methylation of RASSF1A in the serum was detected in 122 of 190 (64.2 %) from patients from the HCC group, in 20 of 114 (17.5 %) patients from the LC group and in 6 of 120 (5.0 %) patients from the CHB group, but no methylation of RASSF1A was detected in the healthy controls. The rate of serum RASSF1A methylation in patients with HCC patients was significantly higher than that in patients with LC or CHB patients (*P* < 0.001) Fig. 1.

### Evaluation of serum RASSF1A methylation as a potential HCC diagnostic marker for HCC

**Fig. 2:**
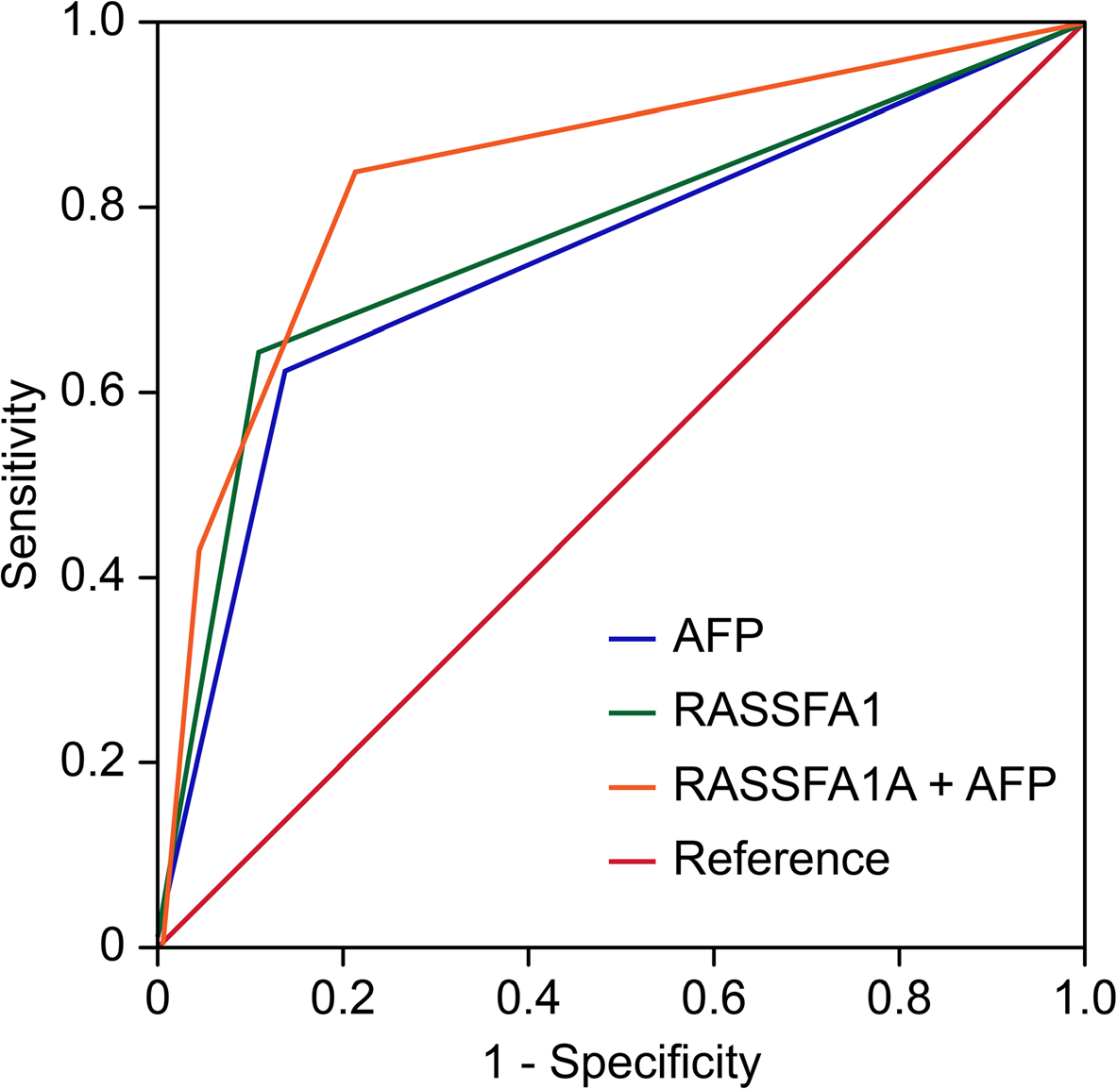
Receiver operating characteristic (ROC) curve analyses using serum RASSF1A methylation and AFP for the discrimination of patients with HCC from patients with CHB. Positive serum RASSF1A methylation yielded an AUC of 0.796 (95 % CI: 0.721–0.864) with a sensitivity of 64.2 % and a specificity of 89.8 % for the discrimination of patients with HCC from patients with CHB; serum AFP at the cut-off value (≥20 ng/mL) yielded an AUC of 0.756 (95 % CI: 0.652–0.805) with a sensitivity of 62.1 % and a specificity of 80.7 %; combined, both indicators yielded an AUC of 0.876 (95 % CI: 0.781–0.933) with a sensitivity of 80.9 % and a specificity of 93.4 %

To further investigate the diagnostic value of serum RASSF1A methylation in HCC, ROC curves were constructed. Serum RASSF1A methylation discriminated HCC patients from CHB patients with an AUC of 0.796 (95 % CI = 0.721–0.864), the sensitivity and specificity for it was 64.2 and 89.8 %, respectively. The serum AFP at the cut-off value of 20 ng/mL yielded an AUC of 0.756 (95 % CI = 0.652–0.805) with a sensitivity of 62.1 % and a specificity of 80.7 %. Furthermore, the AUC for the combination of both indicators was 0.876 (95 % CI = 0.781–0.933), the sensitivity 80.9 % and specificity at 93.4 % Fig. 2.

### Methylation and clinicopathological characteristics in HCC patients

The association between serum RASSF1A methylation and the clinicopathological characteristics of HCC was assessed (Table 2). Some of the clinical parameters, such as histological grading, tumor stage and portal venous invasion were significantly related to serum RASSF1A methylation (*P* < 0.05). However, no significant relationship was observed between serum RASSF1A methylation and the other parameters, such as serum HBeAg status, tumor number, tumor size and liver cirrhosis (*P* > 0.05).

**Table 2:** Correlation between serum RASSF1A methylation status and clinico-pathological features of HCC [*n* (%)]

Characteristics	Case (*n* = 190)	RASSF1A methylation positive (*n* = 122)	*χ* ^2^	*P*
HBeAg				
Positive	96 (50.5)	71 (58.2)	1.757	0.185
Negative	94 (49.5)	51 (41.8)		
Tumor number				
Single	128 (67.4)	84 (68.9)	0.075	0.784
Multiple	62 (32.6)	38 (31.1)		
Tumor size				
< 5 cm	92 (48.4)	50 (41.0)	1.657	0.198
≥ 5 cm	98 (51.6)	72 (59.0)		
Histological grading				
I + II	134 (70.5)	69 (56.6)	6.378	0.012
III + IV	56 (29.5)	53 (43.4)		
TNM stage				
I + II	68 (35.8)	26 (21.3)	7.859	0.005
III + IV	122 (64.2)	96 (78.7)		
Portal venous invasion				
Yes	122 (64.2)	62 (50.8)	5.506	0.019
No	68 (35.8)	60 (49.2)		
Liver cirrhosis				
Yes	118 (62.1)	64 (52.5)	2.844	0.092
No	72 (37.9)	58 (47.5)		

All data are presented as n (%)

*HCC* hepatocellular carcinoma, *HBeAg* HBV e-antigen

### Serum RASSF1A methylation status and survival of HCC patients

**Fig. 3:**
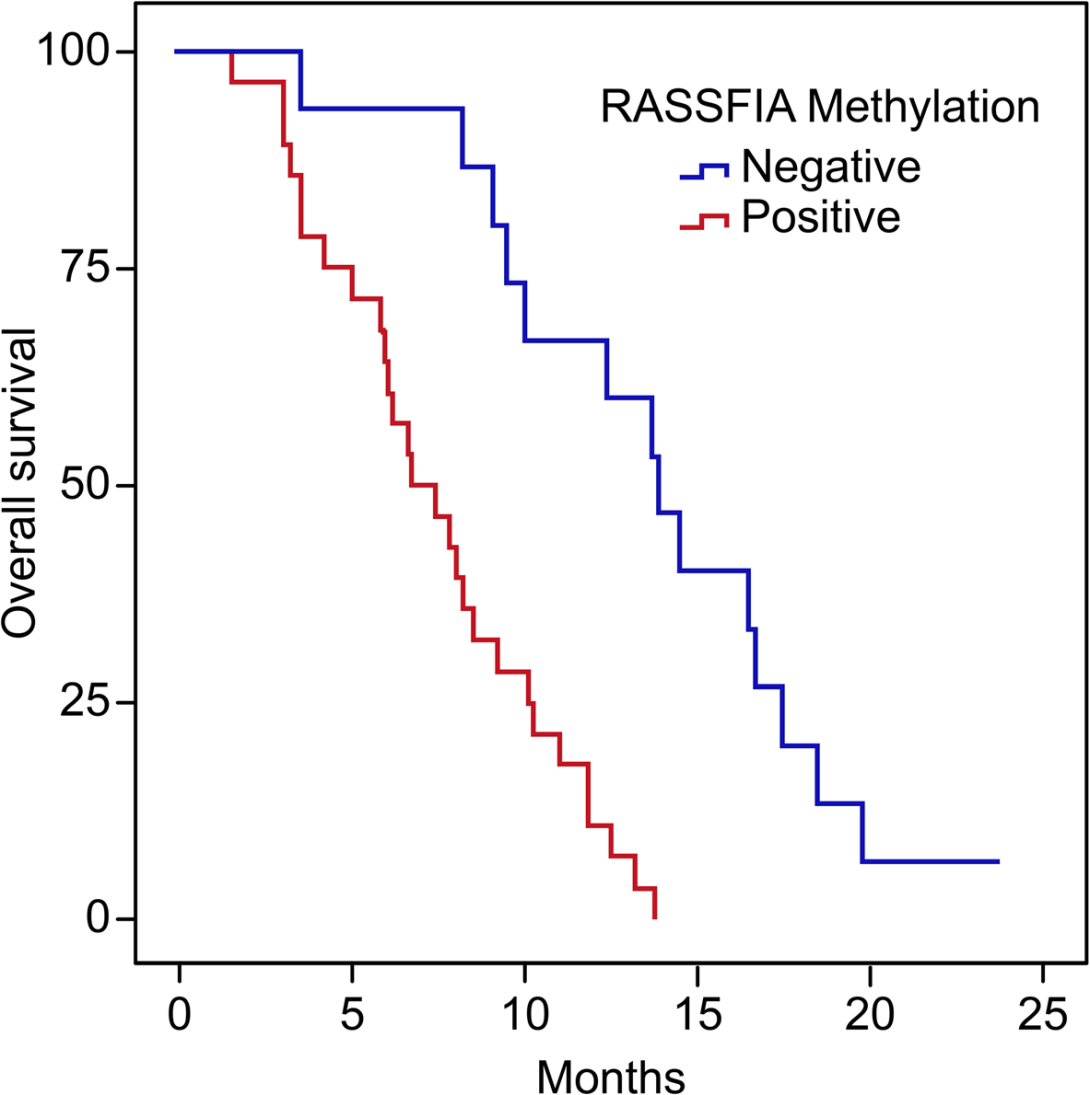
Kaplan–Meier analysis of the overall survival (OS) of 43 patients with HCC. Forty-three patients with HCC were followed-up for 25 months. A Kaplan–Meier analysis showed that patients with HCC with serum RASSF1A positivity (*n* = 24) were more likely to experience a worse OS (*P* = 0.017)

Forty-three HCC patients were unable to undergo surgical operation in this study, which were followed up for 24 months completely. These patients were divided into the positive group (*n* = 24) and the negative group (*n* = 19) according to the PMR of serum RASSF1A promoter. The patients with serum RASSF1A methylation were more likely to have worse OS according to the Kaplan–Meier analysis (*P* < 0.05) (Fig. 3).

## Discussion

Aberrant promoter methylation of the tumor suppressor RASSF1A leads to many malignancies, which indicates that it plays an important role in the development of human cancer [17]. Down-regulation of RASSF1A expression was unrelated to some conventional etiologies, such as HBV / HCV infection, alcohol consumption, and food aflatoxin B1 contamination [3], which suggested that the inactivation of RASSF1A may be a common event in HCC development. Some studies have reported that the rate of RASSF1A methylation was up to 85, 95 and 100 % in HCC tissues, [18–20]. In addition, Hu [21] has shown that reduced of RASSF1A protein expression was related to clinicopathological features of HCC patients with regard to TNM stage, AFP level, tumor metastasis and presence of multiple nodules. These data suggested that RASSF1A methylation may be a promising non-invasive biomarker for HCC. However, the results of current studies on the diagnostic sensitivity and specificity of RASSF1A methylation in peripheral blood of HCC patient have been so varied [9]; this phenomenon may be attributed to the detection methods or the sample size, and therefore its non-invasive application value for HCC needs to be further confirmed.

In this study, we used the MethyLight method to detect serum RASSF1A methylation in a larger sample, and when the PRM ≥4 the result is positive. We found that the rate of serum RASSF1A methylation in HCC patients was significantly higher than that in LC or CHB patients; however, no RASSF1A methylation was detected in healthy subjects. Currently, the serum AFP level (≥20 ng/L) is widely used in the distinction of HCC from CHB, and the diagnostic sensitivity and specificity were 62.1 and 80.7 % in this study, respectively. In the same way, serum RASSF1A methylation yielded a sensitivity of 64.2 % and specificity of 89.8 %, which were prior to AFP level (≥20 ng/L). When RASSF1A methylation and serum AFP are used in combination to distinguish HCC from CHB, the sensitivity and specificity are inceased to 80.9 and 93.4 %, respectively. This result indicates that both indicators combined would be a better marker for HCC in HBV prevalence region. Through an analysis of the relationship between the RASSF1A methylation status and the clinicopathological features of HCC patients, we found that serum RASSF1A methylation was associated with higher histological grading, tumor stage and the occurrence of portal venous invasion. This result suggested that serum RASSF1A methylation positive could predict the clinical progression of HCC patients.

In addition, we conducted 2 years of follow-up study in 43 patients who were not treated with surgery. Our results showed that the OS of patients with serum RASSF1A methylation was significantly lower than that of the patients without RASSF1A methylation. Circulating free DNA may result from the formation of circulating tumor cells or DNA fragments generated by tumor cell necrosis and apoptosis [22, 23]. This hypothesis implies that serum RASSF1A methylation may originate in circulating tumor cells, which then leads to tumor metastasis.

## Conclusion

In conclusion, serum RASSF1A methylation may be a valid biomarker for HBV-related HCC and could predict clinical progression. Specifically, the combination of serum RASSF1A methylation and the AFP level could strengthen the power of discrimination of HCC and CHB.
